# Supplementary material for: Nanoscale Two-Dimensional FeII- and CoII-Based Metal–Organic Frameworks of Porphyrin Ligand for the Photodynamic Therapy of Breast Cancer
Source: Molecules. 2023 Feb 24;28(5):2125. doi: 10.3390/molecules28052125 (PMC10003974; doi:10.3390/molecules28052125)
Supplement: Supplementary file 1 [file molecules-28-02125-s001.zip › Supplementary Material_Revised.pdf]

Supplementary Material for

Nanoscale Two-dimensional Fe^{II}- and Co^{II}-Based Metal−Organic Frameworks of Porphyrin Ligand for the Photodynamic Therapy of Breast Cancer

Qing Li [1,#], Bo-Wei Xu [1,#], Yi-Min Zou [2], Ru-Jie Niu [1], Jin-Xiang Chen [2,*], Wen-Hua Zhang [1,*] and David J. Young [3]

[1] College of Chemistry, Chemical Engineering, and Materials Science, Soochow University, Suzhou 215123, China
[2] NMPA Key Laboratory for Research and Evaluation of Drug Metabolism, Guangdong Provincial Key Laboratory of New Drug Screening, School of Pharmaceutical Sciences, Southern Medical University, Guangzhou 510515, China
[3] College of Engineering, Information Technology & Environment, Charles Darwin University, Darwin, Northern Territory 0810, Australia
[#] These authors contributed equally to this work
* Correspondence: whzhang@suda.edu.cn (W.-H.Z.); jxchen@smu.edu.cn (J.-X.C.)

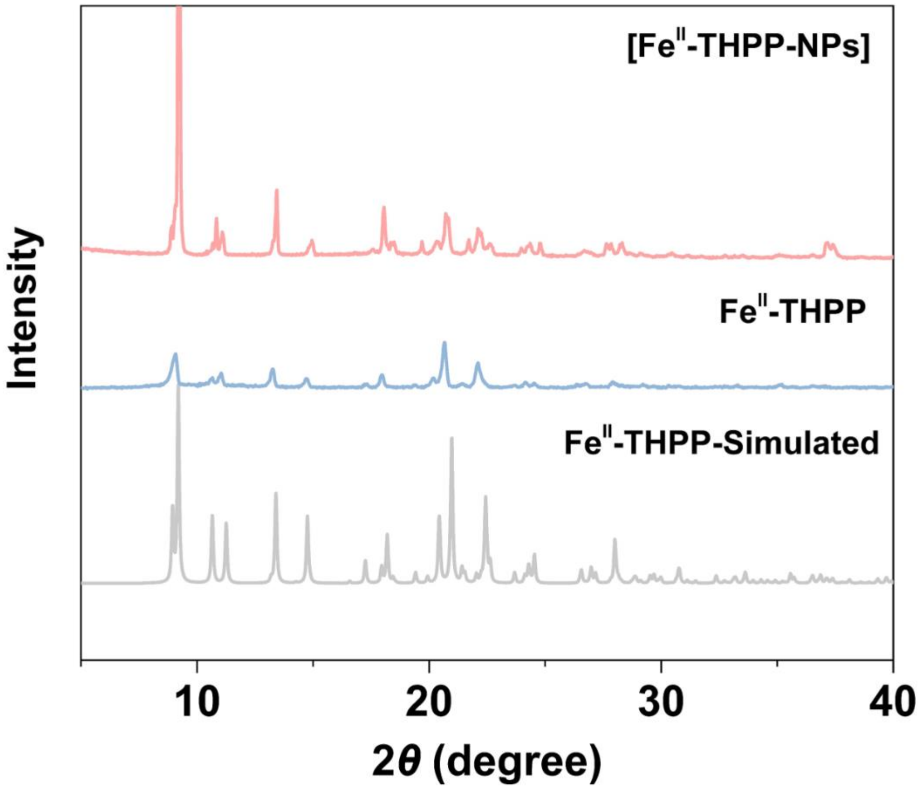

Figure S1. The PXRD patterns of the [Fe^{II}-THPP-NPs] (light-red), Fe^{II}-THPP (experimental, light-blue), and Fe^{II}-THPP (simulated, gray), showing the good consistency of these diffraction patterns and thus the shared structure connectivity of these materials.

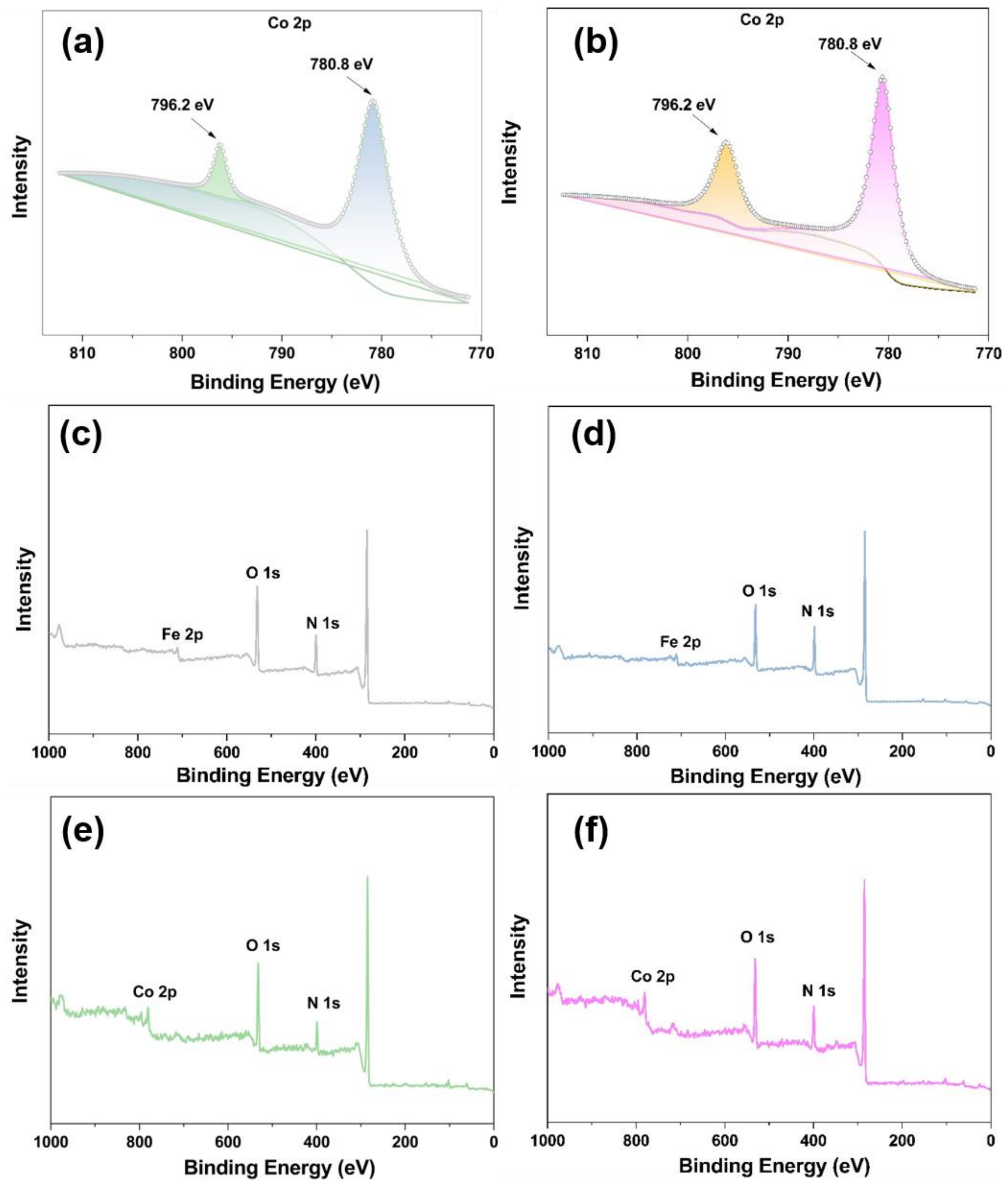

Figure S2. The Co 2p XPS spectra of [Co^{II}-THPP-flower] (a) and [Co^{II}-THPP-film] (b), showing binding energies at 780.8 eV (Co 2p$_{3/2}$) and 796.2 eV (Co 2p$_{1/2}$) (a, b). The full XPS spectra of Fe^{II}-THPP (c), [Fe^{II}-THPP-NPs] (d), [Co^{II}-THPP-flower] (e) and [Co^{II}-THPP-film] (f).

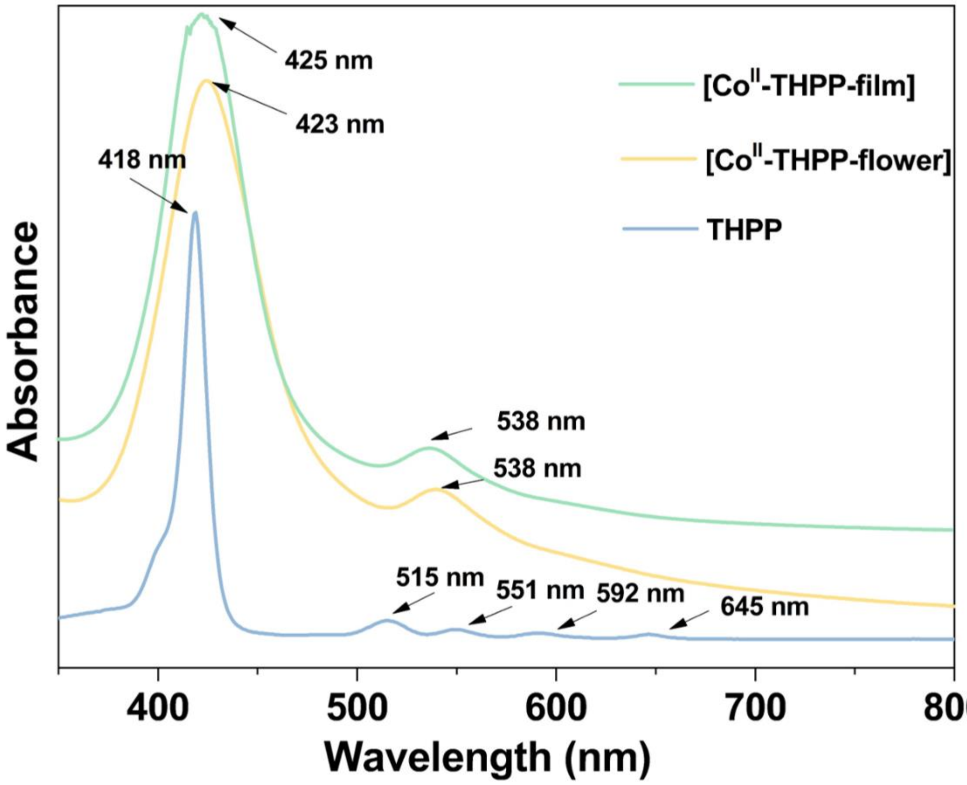

Figure S3. UV-Vis absorption spectra of the free-based ligand THPP (light-blue), [Co^{II}-THPP-flower] (light orange) and [Co^{II}-THPP-film] (light green) in MeOH solution.

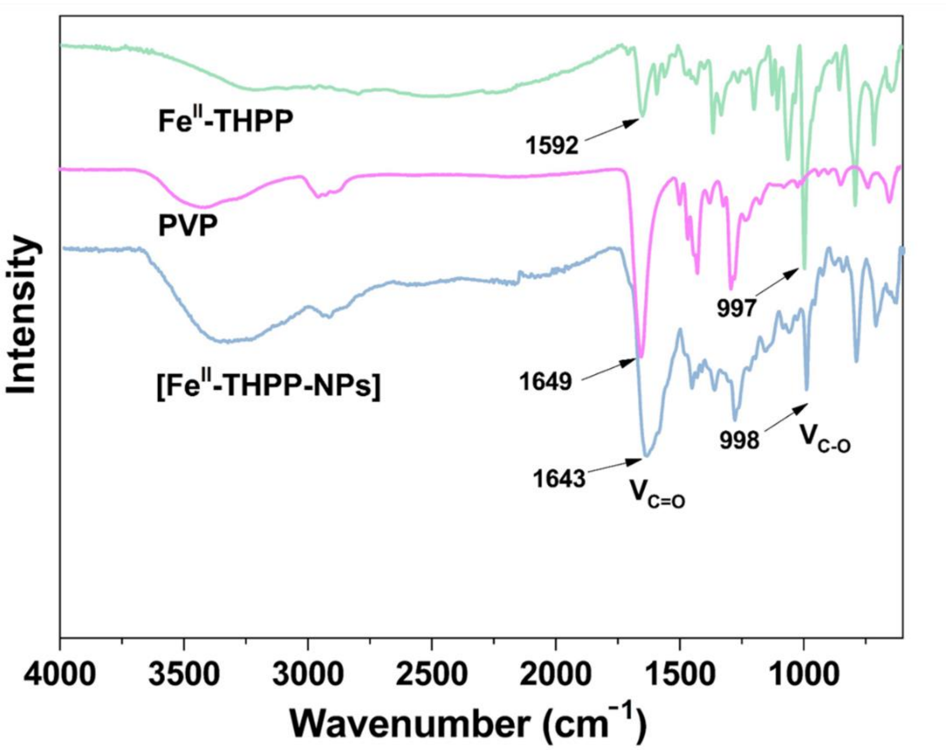

Figure S4. FT-IR spectra of PVP, Fe^{II}-THPP, and [Fe^{II}-THPP-NPs], showing the successful coating of PVP onto the surface of Fe^{II}-THPP.

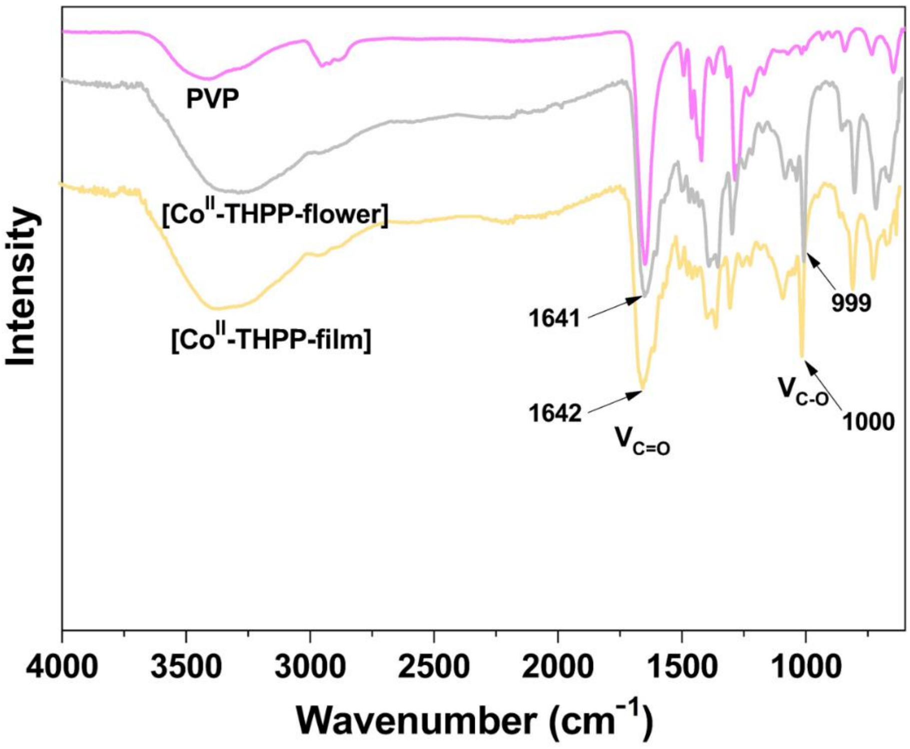

Figure S5. A comparison of the FT-IR spectra of PVP, [Co^{II}-THPP-flower] and [Co^{II}-THPP-film], showing the successful coating of PVP on the surface of [Co^{II}-THPP-flower] and [Co^{II}-THPP-film].

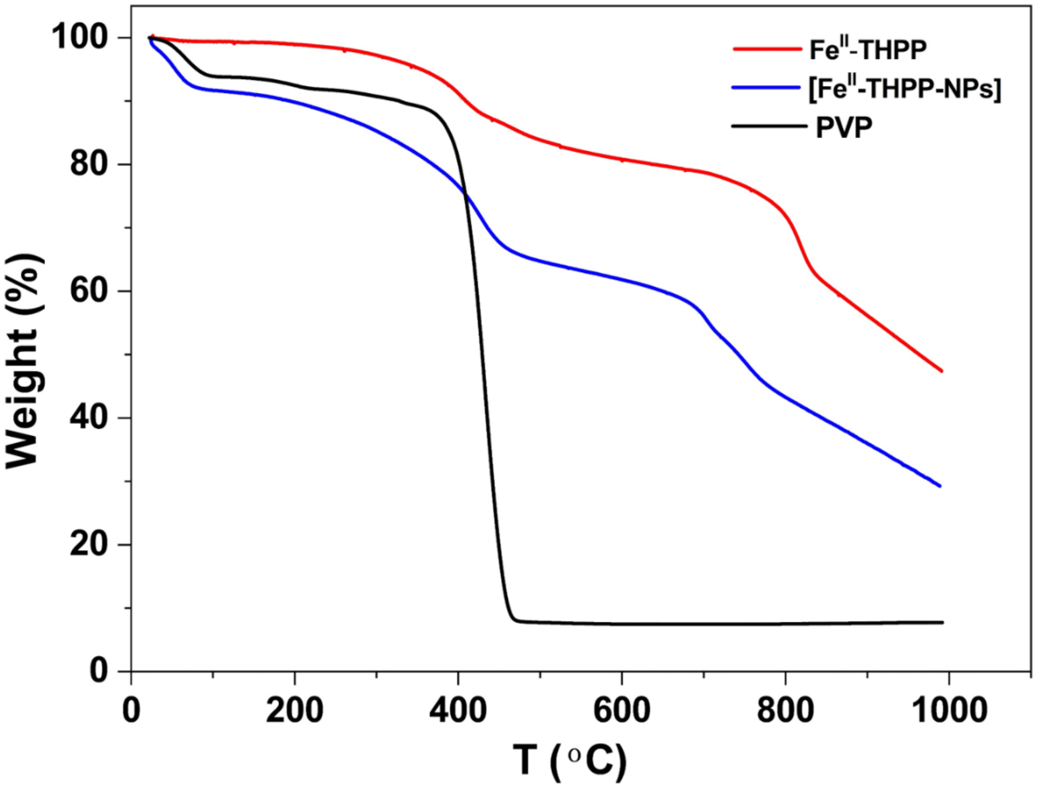

Figure S6. TGA curves of PVP (black), Fe^{II}-THPP (red), and [Fe^{II}-THPP-NPs] (blue) heated up to 1000°C.

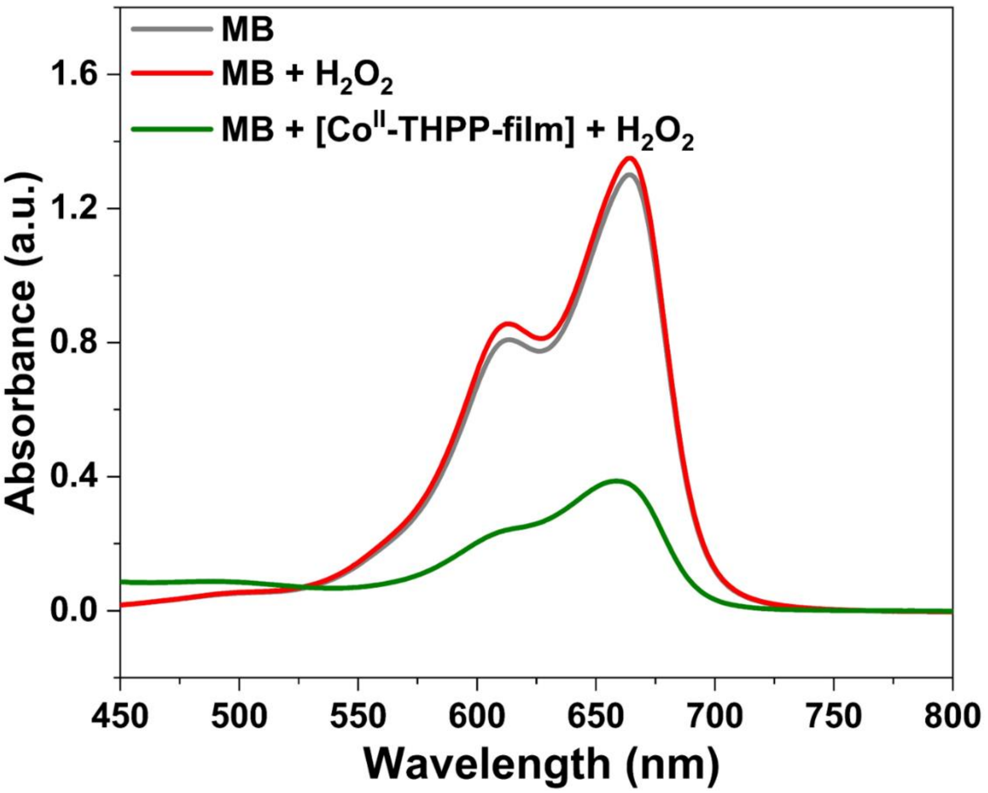

Figure S7. UV-Vis absorption spectra of MB, MB with H_2O_2, and MB with H_2O_2 in the presence of [Co^{II}-THPP-film] after incubation in PBS buffer for 30 min.

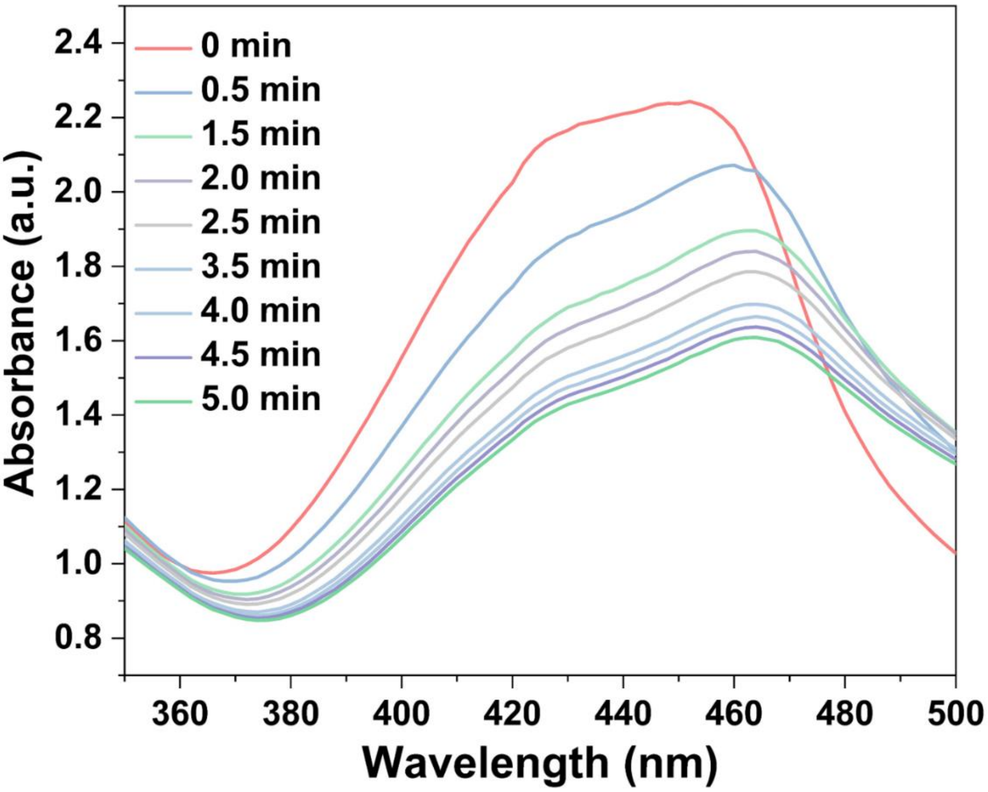

Figure S8. DPBF absorption changes in the presence of [Co^{II}-THPP-film].

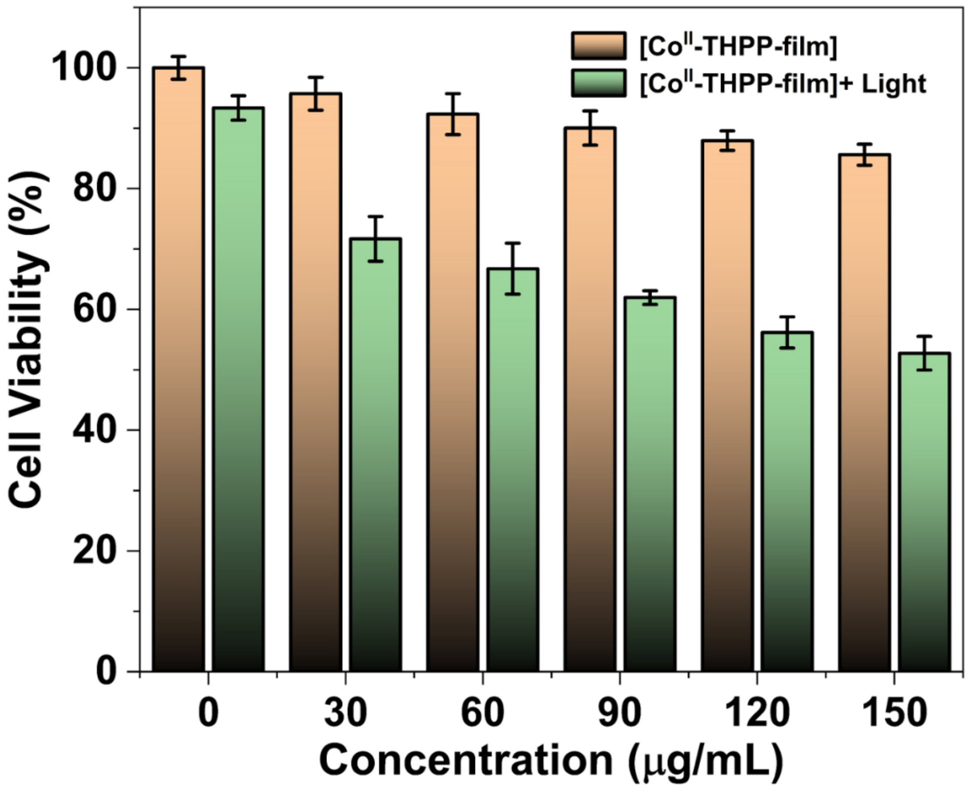

Figure S9. The cell viability of 4T1 cells treated with different concentrations of [Co^{II}-THPP-film] in the absence/presence of 660 nm laser (220 mW cm^{-2}) irradiation. Data are represented as means ± SD; n = 5.
